# Pickering emulsion stabilized by palm-pressed fiber cellulose nanocrystal extracted by acid hydrolysis-assisted high pressure homogenization

**DOI:** 10.1371/journal.pone.0271512

**Published:** 2022-08-31

**Authors:** Shi-Wan Ng, Wai-Ting Chong, Yee-Theng Soo, Teck-Kim Tang, Nur Azwani Ab Karim, Eng-Tong Phuah, Yee-Ying Lee

**Affiliations:** 1 School of Science, Monash University Malaysia, Selangor, Malaysia; 2 School of Food Studies and Gastronomy, Taylor University Lakeside Campus, Selangor, Malaysia; 3 Sime Darby Research Sdn Bhd, R&D Carey Island-Upstream, Selangor, Malaysia; 4 Food Science and Technology, School of Applied Sciences and Mathematics, Universiti Teknologi Brunei, Gadong, Brunei Darussalam; 5 Monash-Industry Palm Oil Education and Research Platform, Monash University Malaysia, Selangor, Malaysia; University of Queensland, AUSTRALIA

## Abstract

Palm pressed fibre (PPF) is a lignocellulose biomass generated from palm oil mill that is rich in cellulose. The present work aimed to combine acid hydrolysis followed by high-pressure homogenisation (HPH) to produce nanocrystal cellulose (CNC) with enhanced physicochemical properties from PPF. PPF was alkaline treated, bleached, acid hydrolysed and homogenised under high pressure condition to prepare CNC. The effects of homogenisation pressure (10, 30, 50, 70 MPa) and cycles (1, 3, 5, 7) on the particle size, zeta potential and rheological properties of CNC produced were investigated. HPH was capable of producing CNC with better stability. Results revealed that utilizing 1 cycle of homogenisation at a pressure of 50 MPa resulted in CNC with the smallest dimension, highest aspect ratio, moderate viscosity and exceptionally high zeta potential. Subsequently, 0.15% (CNC _0.15_ -PE) and 0.30% (CNC _0.30_ -PE) of CNC was used to stabilise oil-in-water emulsions and their stability was evaluated against different pH, temperature and ionic strength. All the CNC-stabilised emulsions demonstrated good thermal stability. CNC _0.30_ -PE exhibited larger droplets but higher stability than CNC _0.15_ -PE. In short, CNC with gel like structure has a promising potential to serve as a natural Pickering emulsifier to stabilise oil-in-water emulsion in various food applications.

## Introduction

Malaysia has emerged as the second largest palm oil producer in the world after its neighbouring country Indonesia. Even so, only 10% oil is extracted from the fruitlet whilst the remaining 90% is discarded as waste [1]. Palm pressed fibre (PPF) is the biomass waste generated from the oil palm fruits after oil extraction. As a lignocellulosic material, it is composed of 33.9% cellulose, 26.1% hemicellulose, 27.7% lignin, 6.9% extractives and 3.5% ash [2, 3]. At present, PPFs are combusted in the boilers at palm oil mills to generate steam and electricity with the balance sold to the open market [4].

Nanocrystal cellulose (CNC) is composed of a highly crystalline phase in which the amorphous regions is eliminated. It has received great attention from the research community due to its strength, modulus, rheology, dimensions and optical properties, or to its renewability, biocompatibility, biodegradability and sustainability [5, 6]. It can be used for various applications such as fabric and textiles, reinforcement in polymer composite, biomedical implants, antimicrobial and transparent films, aerogels, pharmaceuticals, drug delivery, food and cosmetics [7].

Typically, CNC produced are further chemically modified to introduce new functionalities for diverse applications. In the study, esterification and amidation methods have been used to modify the surface of CNC, to prevent aggregation and enhance the dispersion and compatibility of CNC [8]. Both methods involved of massive use of solvents (such as acetone, methyl ethyl, ketone, and tolune) and other chemicals (1-ethyl-3-(3-dimethylaminopropyl) carbodiimide) that can be a threat to human health and the ecosystem. Other than that, esterification method also involved successive solvents exchange process, which is complicated and required long time of processing. Svagan et al. [9] carried out surface modification by covalently cross-linking the nanocellulose layer of oil-in-water Pickering emulsions with aromatic diisocyanate to develop liquid-core capsules with high mechanical stability. The disadvantages of these chemical modification methods are that these chemicals are relatively non environmental-friendly and potentially hazardous, as well as invoved complicated and time consuming process [10].

High pressure homogenisation (HPH) is an effective method in reducing the particle size. Sample is forced through a narrow homogenisation valve at high velocity under pressure which causes the disintegration of large particles into smaller ones. Several studies have proposed the combination of acid hydrolysis and HPH in fabricating nanocellulose particles with better functional properties to circumvent the drawbacks of chemical modifications [11, 12]. Tian et al. [13] reported that CNFs produced by HPH treatment exhibited high viscosity and entangled network structures which is suitable in stabilising Pickering emulsions.

Pickering emulsions are emulsions stabilised by solid particles such as clay, latex, laponite and silica. Advantage of using solid particles is that it can effectively and irreversibly adsorb onto the oil-water interface forming a protective barrier around the droplets through electrostatic repulsion and steric stabilisation which prevent droplet coalescence [14]. Additionally, the particles can form a network in the continuous phase which results in increased viscosity, hence impeding droplet coalescence and retarding creaming or sedimentation of oil droplets [15]. Today, sustainable and food grade solid particles are much sought after to stabilise the Pickering emulsion. Today, many studies had been conducted of using nanocellulose as an excellent Pickering emulsifier [16, 17]. Previous studies showed that Pickering emulsion stabilized by CNC exhibited good surface activity resulting excellent physical stability and desirable functionality [18, 19]. Pickering emulsion stabilized by CNC could remained stable for at least one month and half-life of active component encapsulated in Pickering emulsion was stable and improved under a controlled condition [18].

To the best of our knowledge, there is a limited number of works to produce CNC from PPF using a combination of acid hydrolysis and high-pressure homogenization. Therefore, the main objective of the present work was to explore the feasibility of using simple and productive approach *via* HPH, to enhance the functionalities of the acid-hydrolysed palm-pressed fiber CNC instead of chemicals/solvents that can be used as Pickering emulsifier in accommodating the demand for natural emulsifiers in the food industries. The effects of pressure (10, 30, 50, 70 MPa) and number of cycles (1, 3, 5, 7) of HPH on the physicochemical properties of CNC were investigated. Furthermore, the storage stability of the Pickering emulsion stabilised by 0.15% and 0.30% of CNC against different pH (pH 2, 7, 11), temperatures (4, 25, 90°C) and ionic strengths (0, 50, 125 mM) was assessed over a period of 6–14 days.

## Materials and methods

### Materials

Palm pressed fibre (PPF) and palm kernel olein were supplied by Sime Darby Research Plantation Sdn Bhd (Pulau Carey, Malaysia). Other reagents were: 95.0–98.0 wt% sulfuric acid (Systerm Chemicals, Selangor, Malaysia), 37% hydrochloric acid (Thermo Fisher Scientific, Massachusetts, USA), sodium chloride (Thermo Fisher Scientific, Massachusetts, USA), sodium chlorite (Friendemann Schmidt, Washington, USA), sodium hydroxide (Thermo Fisher Scientific, Massachusetts, USA), potassium hydroxide (Thermo Fisher Scientific, Massachusetts, USA) and 99.5% glacial acetic acid (Thermo Fisher Scientific, Massachusetts USA). Clorox bleach (Clorox, California, USA) was obtained from a local supermarket.

### Preparation and purification of cellulose fibres

#### Cleaning of PPF

Coarse particles such as stones, seeds and leaves were first removed from PPF by hand picking. After that, PPF was soaked in distilled water for 3 hours before being rinsed with hot water (60°C) to wash off dirt adhering to the fibre surface. The cleaned PPF was then dried overnight in an oven at 60°C. The dried PPF was ground with a grinder (Panasonic Mixer Grinder AC-210S, Panasonic, Osaka, Japan) and subsequently sieved with a 60-mesh sieve. Ground PPF was then stored at 4°C until further use.

#### Alkali and bleaching treatments

Ground PPF was subjected to alkali and bleaching treatments in order to remove lignin and hemicellulose. Firstly, ground PPF was treated with 2% (w/v) sodium hydroxide (NaOH) solution at a fibre to solution ratio of 3:50: (g:mL) for 2 h at 80°C under constant mechanical stirring. The alkali treatment was performed two times. After each treatment, the fibre was filtered and washed with distilled water to remove excess alkali. Next, the alkali-treated fibre was added into bleaching solution that was made up of equal parts of 2% Clorox bleach and acetate buffer (2.7 g NaOH and 7.5 mL glacial acetic acid, diluted to 100 mL of distilled water) at a fibre to solution ratio of 3:50 (g:mL). The bleaching treatment was conducted four times at 60°C for 1 h under constant agitation. The bleached fibre was washed with distilled water until neutral pH and stored at 4°C for further use.

### Extraction of cellulose nanocrystals

After completing the chemical treatments, acid hydrolysis was carried out to extract cellulose nanocrystals by dispersing the alkali and bleach treated PPF (ABT-PPF) in 62% w/w sulfuric acid (H_2_SO_4_) in a fibre to solution ratio of 1:15 (g:mL). The hydrolysis process was maintained at 45°C for 30 min under a constant stirring speed. The suspension was diluted to 10-fold with cold distilled water immediately to terminate the reaction. It was then washed with distilled water to reduce the acid concentration and finally neutralised with 0.5 N NaOH solution until a neutral pH was attained.

Ultimately, HPH treatments (PandaPLUS 2000, GEA Group, Düsseldorf, Germany) were applied to further defibrillate the nanocellulose particles. Aliquots of acid hydrolysed cellulose (AH-C) suspension were passed through the HPH at different pressures (10, 30, 50 and 70 MPa denoted as C-10P, C-30P, C-50P and C-70P) for 1 cycle and different homogenisation cycles (1, 3, 5 and 7 times denoted as C-1C, C-3C, C-5C and C-7C) at 50 MPa, respectively. Subsequently, the cellulose suspensions were stored at 4°C until further use.

### Characterization of the cellulose nanocrystals

#### Chemical composition

The chemical compositions of Raw PPF and ABT-PPF were analysed. According to the standard procedures described by Then et al. [20], the holocellulose (α-cellulose + hemicellulose) content was estimated by treating the fibre with 5 wt% sodium chlorite (NaClO_2_) solution using a fibre to liquor ratio of 1:20 (g/g). The mixture was adjusted to pH 4 using H_2_SO_4_ solution and heated to 70°C for 1 h. The residue was filtered, washed and oven-dried at 60°C until a constant weight was achieved. To determine the cellulose content, the holocellulose was treated with 6 wt% potassium hydroxide (KOH) solution using a fibre to liquor ratio of 1:20 (g/g). The reaction was allowed to stand for 24 h at 25°C. The residue was filtered, washed and oven-dried at 60°C until a constant weight was achieved. The hemicellulose content was calculated as the difference between holocellulose and cellulose contents of the fibres. The lignin content was also measured following the standard method of Technical Association of Pulp and Paper Industry TAPPI T222 om-02. Briefly, 1 g of each sample was treated with 15 mL 72% H_2_SO_4_ at 30°C for 1 h, followed by dilution to 3% acid concentration and boiled for 4 h. The residue was filtered, washed and oven-dried at 60°C to constant weight. Lignin content was calculated according to the following formula:

$$Lignin\;(\%)=\frac{A}{W}\times 100$$

where A is the weight of lignin in grams and W is the oven-dry weight of sample in grams.

#### Particle size and zeta potential

The particle size and zeta potential of the samples were measured using Malvern Nano-ZS Zetasizer (Malvern Instruments Ltd, UK). Dynamic light scattering (DLS) method was adopted to evaluate the particle size distribution whereas the zeta potential of samples was measured by principle of electrophoretic light scattering (ELS). Prior to measurements, the samples were diluted 100 times using sodium chloride (NaCl) solutions (20 mM) to avoid multiple scattering effects. Aspect ratio was calculated later at the ratio of length to diameter.

#### Field emission scanning electron microscopy (FE-SEM)

FE-SEM was performed to examine the morphology of Raw PPF, ABT-PPF, AH-C and high pressure homogenised CNC (HPH-CNC). Samples were fixed on an aluminium stub using carbon filled tape and then coated with gold in a sputter coater (Quorum Q150R S, Quorum Technologies, Lewes, UK) at sputter current 30 mA, sputter time 38 seconds and tooling factor 1.10 in order to increase conductivity. The coated samples were visualized under a scanning electron microscope (Hitachi SU8010, Tokyo, Japan) operating at 5 or 10 kV accelerating voltage. Particle dimension was determined by digital image analysis (ImageJ software).

#### Transmission electron microscopy (TEM)

TEM was performed to observe morphology of HPH-CNC extracte

from PPF. CNC suspension was diluted 100 times using deionized water and then sonicated for 20 min. A drop of CNC suspension was deposited on a copper grid and allowed to dry at room temperature. The images were obtained and recorded using a transmission electron microscope (FEI Tecnai G2 20 S-Twin, Oregon, USA).

#### X-ray diffraction (XRD)

XRD measurements were performed to evaluate the crystalline structure of Raw PPF, ABT-PPF, AH-C and HPH-CNC using an X-ray diffractometer (Bruker D8 Discover, Massachusetts, USA). The diffraction data were collected over a scan diffraction angle varied from 5° to 95° 2θ angular range with a scan rate of 1° min^−1^ at room temperature using an X-ray diffractometer (Bruker D8 Discover, Massachusetts, USA). The equipment was operated at a voltage of 40 kV and a current of 40 mA with the radiation generated by Cu-Kα (wavelength = 1.542 Å). The crystallinity index (CrI) of cellulose was calculated based on the Segal’s empirical method [21], with the following equation:

$$CrI\;(\%)=\frac{I_{002}-I_{am}}{I_{002}}\times 100$$

where *I*_*200*_ represents the peak intensity of crystalline region (2θ = 22°) and *I*_*am*_ represents the peak intensity of amorphous region (2θ = 18°).

#### Fourier transform infrared (FTIR) spectroscopy

FTIR was used to study the changes in functional groups induced by various treatments. The FTIR spectra were acquired in the infrared region ranging from 400 to 4000 cm^-1^ with a resolution of 4 cm^-1^ and a total of 16 scans for each sample using an infrared Fourier-transform spectrometer (Perkin Elmer Spectrum Two, Perkin Elmer, Massachusetts, USA).

#### Rheology

The rheological properties of the HPH-CNC suspensions as a function of homogenisation pressure and cycle were measured at 25°C using a modular compact rheometer (MCR 302, Anton Paar, Graz, Austria) equipped with a parallel plate (50 mm diameter and 1 mm gap). For steady flow test, the shear stress was measured with the shear rate ranging from 0.01 to 300 s^-1^ and the flow curves were obtained in sequential two flow steps: up-down cycles. The second (downward) flow curve data were analysed using the Hershel-Bulkley model as follows:

$$\sigma =\sigma_{0}+K\gamma^{n}$$

where, σ is the shear stress (Pa), σ_0_ is the yield stress (Pa), γ is the shear rate (s^−1^), K is the consistency coefficient (Pa·s^n^), and n is the flow behaviour index (dimensionless).

### Preparation of Pickering emulsions

Aqueous suspensions of HPH-CNC at 1 cycle and 50 MPa was used to prepare Pickering emulsion at a concentration of 0.15 and 0.30% w/w, respectively. The emulsions were formulated with 20% w/w palm kernel olein as the oil phase and 80% w/w CNC aqueous suspensions. The Pickering emulsions were prepared following the method described in study by Soo et al. [22]. Emulsions were produced by using a two-step procedure with pre-mixing followed by homogenisation. The coarse emulsions were homogenized by rotor-stator homogeniser (IKA® T25 digital Ultra-Turrax®, IKA, Staufen, Germany) at 10,000 rpm for 2 min. Secondary emulsions were prepared by passing the coarse emulsions through a HPH (PandaPLUS 2000, GEA Group, Düsseldorf, Germany) for 3 cycles at 20 MPa. Emulsions were stored for 6–14 days to determine their storage stability.

#### Effect of pH on emulsion properties

Stability of the emulsion system against different pH was evaluated. Each sample was adjusted to three different pH, namely pH 2 (acidic), pH 7 (neutral) and pH 11 (alkaline) with 0.1M NaOH or 0.1M HCl solution. Ionic strength and temperature of these samples were kept at 0 mM NaCl and 25°C, respectively.

#### Effect of temperature on emulsion properties

Stability of the emulsion system against different temperatures was evaluated. Each sample was subjected to three different temperatures including 4°C, 25°C and 90°C. The samples were placed at respective places in the refrigerator, workbench and water bath for 30 min. Other parameters such as pH and ionic strength of these samples were kept at pH 7 and 0 mM NaCl, respectively.

#### Effect of ionic strength on emulsion properties

Stability of the emulsion system against different ionic strength was examined. The samples were treated with NaCl which is the most commonly used salt in food industries. The ionic strength of the samples was controlled at 0, 50 and 125 mM by adding the proper amount of NaCl. pH and temperature of these samples were kept at pH 7 and 25°C respectively

### Characterization of emulsions

#### Droplet size determination

The droplet size of the emulsions was determined using a laser diffraction particle size analyser (PSA 1190, Anton Paar, Graz, Austria) equipped with a wet dispersion unit. The sample solution was dispersed in distilled water drop by drop until an obscuration rate of 15–20% was obtained. Refractive index of dispersed phase (palm kernel olein) and continuous phase (water) was set at 1.4578 and 1.33, respectively. Droplet size distribution was measured based on light scattering theory. Droplet size was reported as the volume-weighted mean diameters (d_43_), defined by the following equation:

$$d_{43}=\frac{\Sigma n_{i}d_{i}^{4}}{\Sigma n_{i}d_{i}^{3}}$$

where n_i_ is the droplets number with diameter d_i_.

#### Visual assessment of creaming

The emulsion storage stability against creaming was ascertained by measuring the extent of the gravitational phase. Measurements were performed throughout the 14 days of storage by tracking the total emulsion height (H_T_) and the serum layer height (H_s_) at Day 0, 1, 3, 6 and 14. The extent of creaming was reported as creaming index (CI), calculated with the formula below:

$$CI\;(\%)=\frac{H_{s}}{H_{T}}\times 100$$

#### Optical microscopy

The morphology of oil droplets in emulsions was observed under an optical microscope (Leica DM750, Leica Microsystems GmbH, Wetzlar, Germany) at 40× magnification. The images were acquired using a Leica ICC50W digital camera (Leica).

### Statistical analysis

All measurements were performed in triplicate for each sample unless otherwise stated. Results are expressed as means ± standard deviations. Statistical analysis was carried out with Statistical Package for the Social Sciences (SPSS) 19.0 software. Analysis of variance (ANOVA) is employed to determine significant differences at p < 0.05 using post-hoc least significant difference (LSD) test. Independent t-test was used to determine the statistical difference between the means of two independent variables for emulsion droplet diameter between CNC_0.15_-PE and CNC_0.30_-PE.

## Results and discussion

### Isolation of cellulose nanocrystals

#### Purification and chemical composition

The chemical compositions of Raw PPF, ABT-PPF were determin and presented in Table 1. The Raw PPF consisted of 34.34 ± 0.37% cellulose, 26.82 ± 0.11% hemicellulose and 35.34 ± 0.01% lignin. After alkaline and bleaching treatments, the contents of cellulose, hemicellulose and lignin were 57.17 ± 0.04%, 14.11 ± 0.17% and 13.70 ± 0.16%, respectively. Alkali treatment resulted in the elimination of hemicellulose while bleaching led to the removal of phenolic or chromophores compounds to achieve a whitening effect [23]. The cellulose, hemicellulose and lignin contents of the PPF before pre-treatment in our study were similar to those reported in the previous studies: 21.00–44.00% cellulose, 23.00–38.20% hemicellulose and 17.28–33.00% lignin [24–27]. After pre-treatments, the composition of cellulose, hemicellulose and lignin in these studies changed evidently in the range of 37.70–66.00%, 14.40–34.67% and 4.90–25.00%. The cellulose, hemicellulose and lignin of the present results also fell within the range of these studies. Variation in composition of the treated PPF may be due to different chemicals and methods being used to pre-treat PPF. For example, Lin et al. [23] used hydrogen peroxide and enzyme to pre-treat PPF, Pereira compared the different methods to delignify PPF while Aziz et al. [22] used chlorite and sodium hydroxide to pre-treat PPF. In the present study, there was still some residual lignin present after bleaching. Several studies showed that lignin may serve to attain certain health benefits attributed to its antioxidative properties [28]. Subsequent acid hydrolysis and high pressure homogenization resulted in the production of CNC having 61.92 ± 1.75% of cellulose, 15.36± 0.43% of hemicellulose and 14.61 ± 1.03% of lignin.

**Table 1:** Chemical compositions of Raw PPF and ABT-PPF.

Sample	Raw PPF (%)	ABT-PPF (%)	CNC (%)
Cellulose	34.34 ± 0.37^a^	57.17 ± 0.04^a^	61.92 ± 1.75^a^
Hemicellulose	26.82 ± 0.11^b^	14.11 ± 0.17^b^	15.36 ± 0.43^a^
Lignin	35.34 ± 0.01^c^	13.70 ± 0.16^c^	14.61± 1.03 ^d^

Averages are means of two determinations ± standard deviation.

^abc^ represents the values within the same row with same letters were significantly different (p < 0.05) from each other. ABT-PPF = Alkali and bleach treated PPF. CNC = cellulose nanocrystal

#### Influence of homogenisation on isolation of CNC

Table 2 represents the physical properties of cellulose particles after acid hydrolysis and HPH treatments. It was noted that the cellulose particles obtained by acid hydrolysis exhibited size of 46 μm in length. According to previously published work, CNCs of asparagus spears and stems possessed a length of 189 nm, CNCs of coconut husk fibres had lengths ranging from 80 to 500 nm and CNC of PPF had a mean length of 171 ± 15 nm, respectively [29–31]. The discrepancy could be attributed to the difference in the structure of precursor material, hydrolysis conditions, agglomeration of CNC and incomplete removal of impurities during the pre-treatment process [32]. Studies supported by Ni, Li and Fan [11] also obtained micron-size cellulose particles (14 μm) from ginkgo seed shells after acid hydrolysis. As evidenced by the micrograph, the micron-size CNC could be due to the aggregation fibril bundles. The aggregation of particles could be attributed to the incomplete removal of impurities such as hemicellulose and lignin during the pre-treatment process [32]. It is postulated that inadequate hydrolysis or the peeling of small segments of the fibril during hydrolysis may increase the width and height of CNC, hence producing CNCs with multiple elementary crystals [33]. CNCs strongly agglomerate due to the large freedom of motion after hydrolytic cleavage giving an apparent increase in particle size.

**Table 2:** The length, diameter, aspect ratio and zeta potential of CNC treated by different homogenisation pressures and cycles.

Treatment	Samples	Length (nm)	Diameter (nm)	Aspect ratio	Zeta potential (mV)
Untreated	AH-CNC	46,357.3 ± 2152.3	-	-	–13.4 ± 0.3
Pressure (MPa)	C-10P	474.1 ± 163.9^a^	107.3 ± 28.1^a^	4.38 ± 0.54^a^	–37.3 ± 1.9^b^
	C-30P	439.5 ± 88.8^a^	111.0 ± 22.6^a^	3.98 ± 0.36^a^	–57.6 ± 0.5^a^
	C-50P	362.0 ± 14.4^a^	82.6 ± 6.3^a^	4.39 ± 0.19^a^	–60.6 ± 4.3^a^
	C-70P	374.3 ± 90.0^a^	90.8 ± 20.0^a^	4.11 ± 0.18^a^	–58.9 ± 2.1^a^
Cycle	C-1C	320.5 ± 33.9^A^	89.5 ± 7.5^A^	3.58 ± 0.08^AB^	–62.2 ± 1.7^B^
	C-3C	352.2 ± 74.8^A^	105.2 ± 14.0^AB^	3.33 ± 0.32^AB^	–63.1 ± 1.9^B^
	C-5C	340.0 ± 32.7^A^	110.9 ± 27.6^AB^	3.14 ± 0.44^A^	–64.4 ± 2.0^B^
	C-7C	475.2 ± 71.3^A^	123.7 ± 16.2^B^	3.85 ± 0.33^B^	–69.1 ± 2.6^A^

Averages are means of three determinations ± standard deviation.

^abc^ represents the significance between samples at different pressures; ABC represents the significance between samples at different number of cycles. Aspect ratio was calculated based on the ratio of length to diameter. AH-CNC = Acid hydrolysed CNC; C-10P = CNC treated at 10 MPa; C-30P = CNC treated at 30 MPa; C-50P = CNC treated at 50 MPa; C-70P = CNC treated at 70 MPa; C-1C = CNC treated for 1 cycle; C-3C = CNC treated for 3 cycles; C-5C = CNC treated for 5 cycles; C-7C = CNC treated for 7 cycles.

Therefore, HPH was applied after acid hydrolysis to further reduce the particle size of cellulose particles. Size of cellulose particles dramatically reduced from micron size to nanoscale with 362.0 ± 14.4 nm in length after 50 MPa treatment. An increase in the magnitude of disruptive energy generated by the homogeniser at higher pressure resulted in a greater particle size reduction. This was consistent with some previous reports [11, 34, 35]. For instance, Svagan et al. [9] applied HPH treatment to produce CNC from ginkgo seed shells and reported that the decrease of mean particle sizes was more pronounced at higher homogenisation pressure, particularly in terms of the CNC length.

In terms of homogenisation cycles, the mean particle size of CNC exhibited a significant reduction after 1 cycle of HPH treatment, presenting a length of 320.5 ± 33.9 nm after 1 cycle of HPH. Nevertheless, the particle size of CNC shifted slightly to a higher value after 3, 5 and 7 cycles. Whilst, different pressures at 10, 30, 50 and 70 MPa dramatically reduced the size of cellulose particles from micron size to nanoscale with 474.1 ± 163.9 nm in length and 107.3 ± 28.1 nm in width after 10 MPa treatment. At 30 MPa, CNC displayed an average length of 439.5 ± 88.8 nm and diameter of 111.0 ± 22.6 nm. With increasing homogenization pressure to 50 MPa, the length and diameter of CNC reduced to 362.0 ± 14.4 nm and 82.6 ± 6.3 nm respectively. A further increase of the pressure from 50 to 70 MPa resulted in a slight increase in CNC particle size with 374.3 ± 90.0 nm in length and 90.8 ± 20.0 nm in width. This outcome was inconsistent with previous studies which discovered that the particle size of cellulose particles decreased with increasing homogenisation cycles [11, 14, 36]. However, the result obtained in this study was explainable. The particle size increased as the pressure or cycle increased which could be due to droplet polymerization or re-polymerization [37]. Pelissari et al. [38] who reported that the large freedom of motion after mechanical treatment favoured the growing of the size of cellulose crystallites, hence giving rise to the larger dimensions of the homogenised nanocellulose. This explanation was further supported by Lenhart et al. [39] who found that higher number of cycles led to larger MCC particles due to a tendency to re-agglomerate. Recent studies investigated on the use a combination of enzymatic or acid hydrolysis coupled with high-pressure homogenization in the production of CNC also found to have slightly different results from the current study. In the previous studies, the parameters of HPH used were more extreme which ranges from 60–300 MPa and 6–20 cycles [13, 40, 41]. Therefore, it could be explained that no further reduction in particle size with the increase in pressure and number of cycles applied in this study might be due to the insufficient pressure and cycle. Besides, particle size obtained from dynamic light scattering (DLS) is a relative assessment even though it has been used in many studies [42–46].

**Fig 1:**
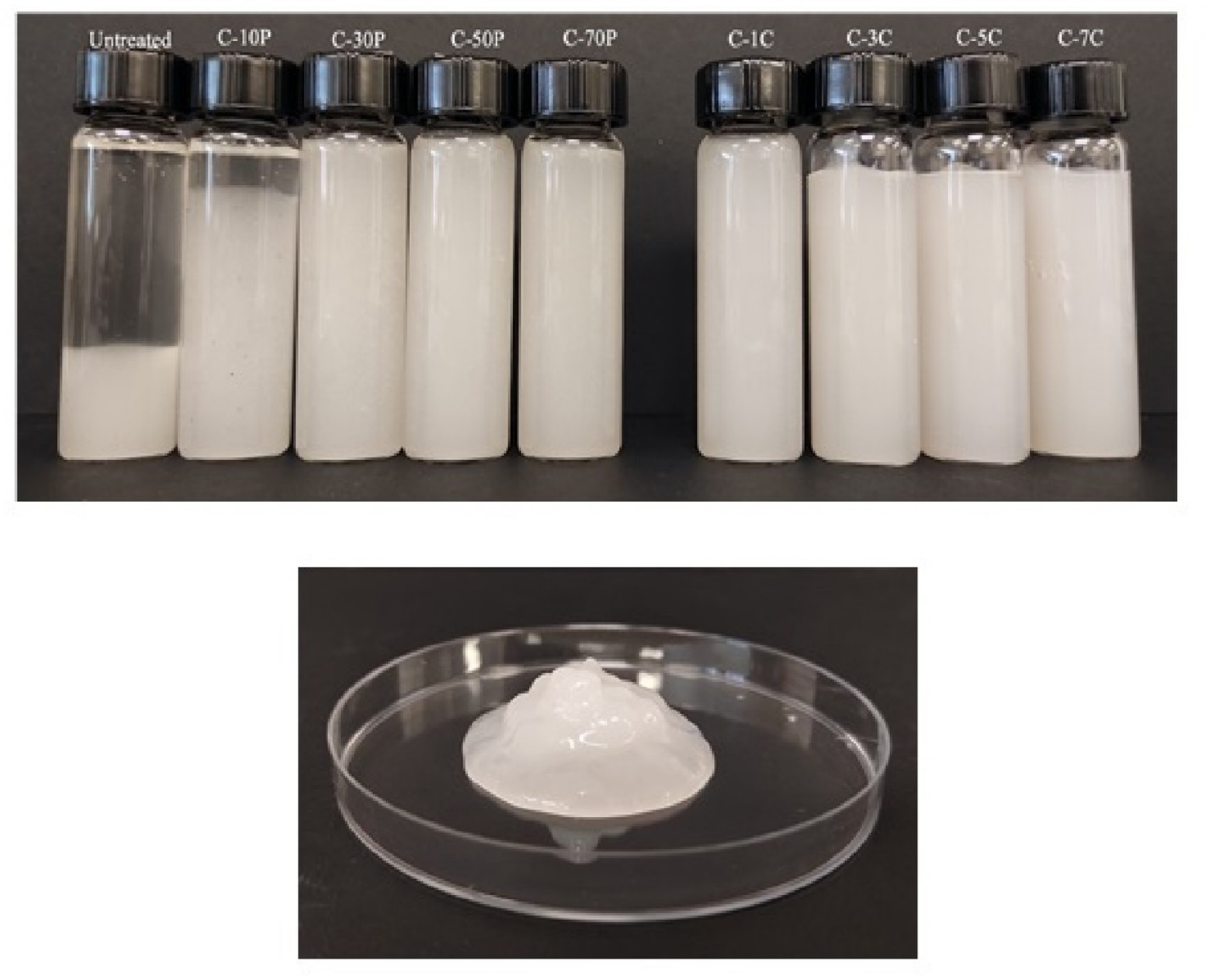
Pictures of (a) CNC suspensions subjected to different HPH treatments (C-10P to C-70P and C-1C to C-7C) and (b) gel-like structure of C-7C sample.From left to right, Untreated = Acid hydrolysed cellulose; C-10P = CNC treated at 10 MPa; C-30P = CNC treated at 30 MPa; C-50P = CNC treated at 50 MPa; C-70P = CNC treated at 70 MPa; C-1C = CNC treated for 1 cycle; C-3C = CNC treated for 3 cycles; C-5C = CNC treated for 5 cycles; C-7C = CNC treated for 7 cycles.

Cellulose particles presented a zeta potential value of −13.4 ± 0.3 after acid hydrolysis. Hydrolysis by sulfuric acid induced the grafting of negatively charged sulfate groups (−OSO_2_O−) on the surface of CNC. Zeta potential of CNC suspensions increased sharply to −37.3 ± 1.9 as homogenisation pressure reached 10 MPa and further increased to −58.9 ± 2.1 with the increase of homogenisation pressure to 70 MPa. Likewise, increasing in the number of passes furnished more stable CNC suspensions with zeta potential values ranging from −62.2 ± 1.7 to −69.1 ± 2.6. HPH treatment promotes the agitation of suspension and increase the contact between CNCs and oxygen, leading to partial oxidation of the particles which generates more negative charge [47]. A similar increase of zeta potential values from −12.3 mV to −50 mV had also been reported for CNC suspensions extracted from ginkgo seed shells after HPH treatment [11]. Interestingly, the CNC produced in this experiment exhibited a higher zeta potential value which was around −60 mV with respect to those reported by Ni *et al*. (2020). The zeta potential values of the CNC corroborate well with the gel-like material forms after HPH as depicted in Fig 1. Prior to homogenisation, the water-fibre suspension obtained by acid hydrolysis showed rapid sedimentation presenting a two-phase nature. HPH treatment transformed the CNC suspensions into gel-like structures which were stable and did not flocculate or settle down [48].

**Fig 2:**
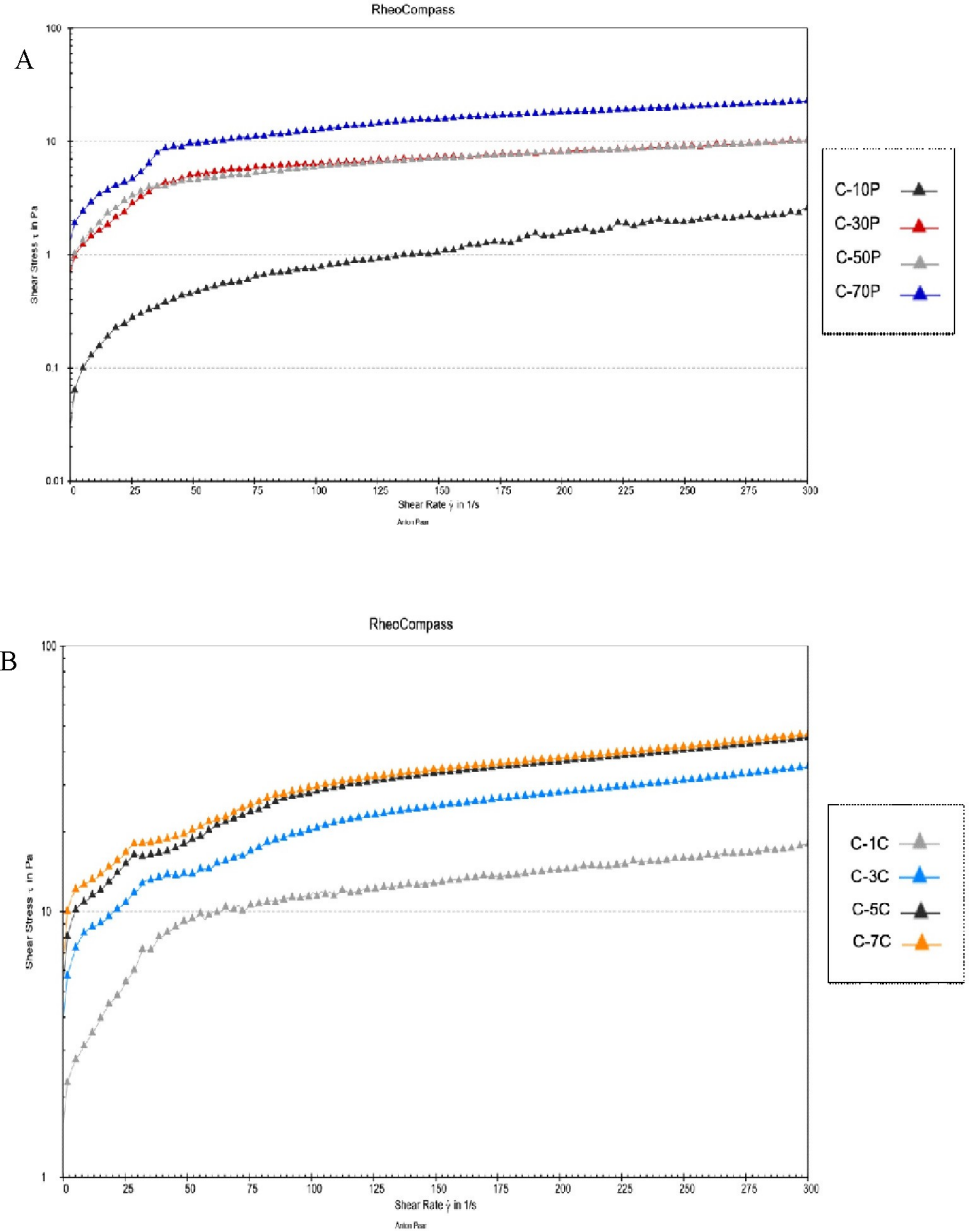
(A) Influence of homogenisation pressure on flow curve of stress as a function of shear rate and (B) Influence of homogenisation cycles on flow curve of stress as a function of shear rate. C-10P = CNC treated at 10 MPa; C-30P = CNC treated at 30 MPa; C-50P = CNC treated at 50 MPa; C-70P = CNC treated at 70 MPa; C-1C = CNC treated for 1 cycle; C-3C = CNC treated for 3 cycles; C-5C = CNC treated for 5 cycles; C-7C = CNC treated for 7 cycles.

The rheological properties of cellulose suspensions as a function of homogenisation pressure or cycle were determined as shown in Fig 2. The Herschel-Bulkley model was applied to fit the stress-shear rate curves. All suspensions exhibited shear-thinning behaviour. As shown in Fig 2, the shear stress increased with the increase of homogenisation pressure and homogenisation cycles. These results manifested that higher homogenisation pressure or number of cycles produced CNC slurry with higher viscosity.

High pressure homogenization provides a large pressure to break down acid-hydrolysed cellulose into smaller and finer fragments. After CNC passing through the HPH, a gel-like suspension is formed with an obvious increase in the viscosity mainly due to the formation of fiber network and interfibrillar force [49]. Defibrillation increases the relative bonded area as there are more CNC interacts with water and more entanglement of the cellulose to entrap water. This gives a clear explanation for the reason as to no further reduction in particle size was observed, but viscosity increased dramatically when higher homogenization pressure and cycle applied. The increase in interfibrillar force and entanglement of cellulose contribute to the rise in viscosity.

#### XRD

**Fig 3:**
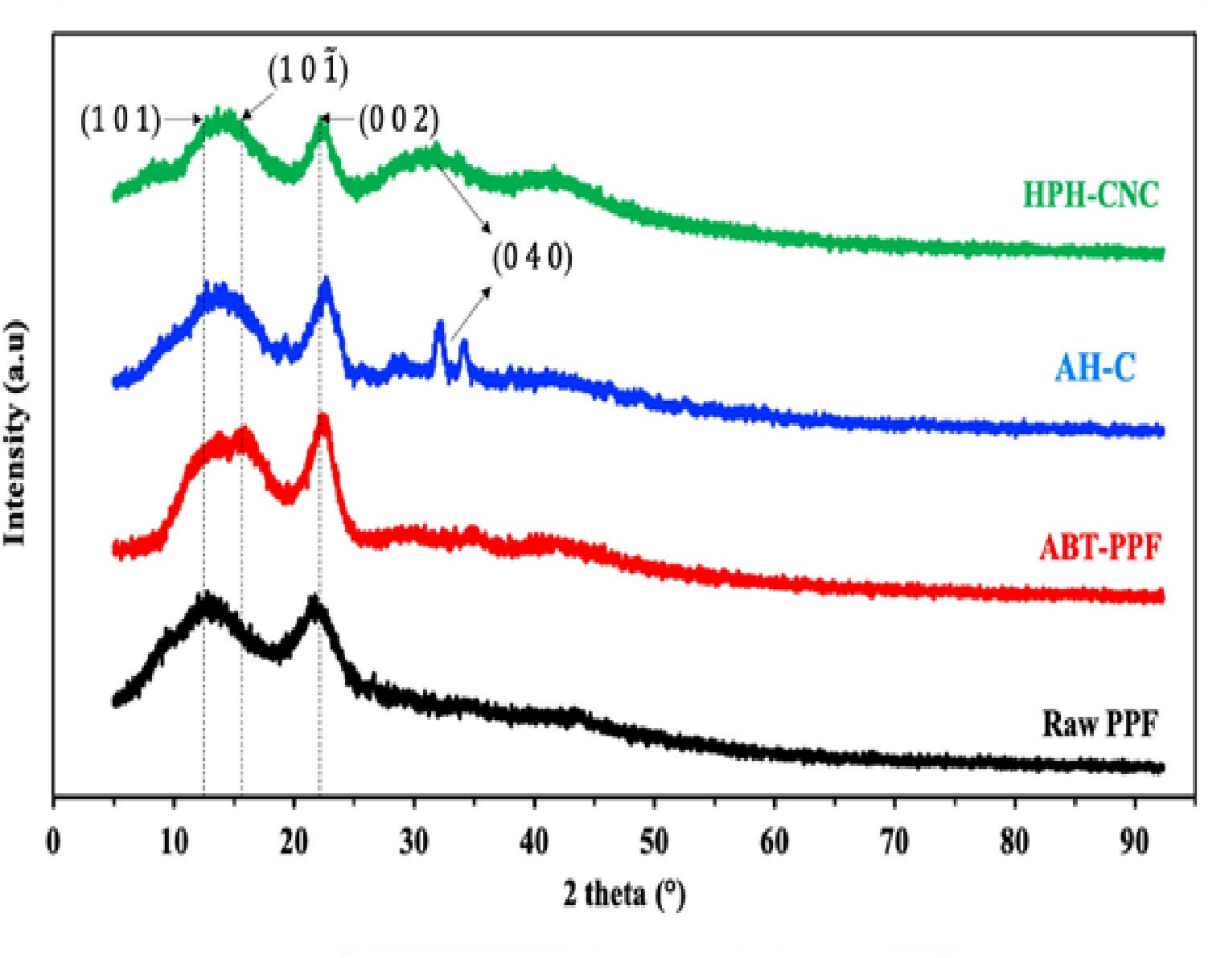
XRD diffractograms of Raw PPF, treated PPF, AH-C and HPH-CNC.

Fig 3 shows the X-ray diffractograms whilst Table 3 demonstrates the crystallinity index of the Raw PPF, ABT-PPF, AH-C and HPH-CNC. Raw PPF exhibited the peaks that were broad and low due to the presence of non-crystalline hemicellulose, lignin and pectin. After alkaline and bleaching treatments, the removal of hemicellulose and lignin contributed to the narrowing of the diffraction peaks and increased the crystallinity of fibres from 30.35% to 39.19%. Acid hydrolysis increased the CrI to 56.82% due to break down the glycosidic bonds in the amorphous regions by hydronium ions and realignment of monocrystals. The improvement of crystallinity after acid hydrolysis was also evidenced by the development of new crystalline peaks at 2θ = 32° and 34° corresponding to (0 4 0) crystallographic planes that reflected cellulose I [50]. However, after HPH treatment, the CrI decreased to 44.85%. Similar results were also observed for the extraction of CNC from ginkgo seed shells [11], microfibrillated cellulose (MFC) from mangosteen rind [14] and sugarcane bagasse [51] by HPH. It is postulated that the intense HPH treatment imparted high shear forces on the crystal surface, causing the partial destruction of intermolecular and intramolecular hydrogen bondings of cellulose which resulted in the collapse of crystal structure.

**Table 3:** Crystallinity index of Raw PPF, ABT-PPF, AH-CNC and HPH-CNC.

Sample	2θ (Amorphous) (°)		2θ (002) (°)		CrI (%)
	Degree	Intensity (I_am_)	Degree	Intensity (I_002_)	
Raw PPF	18.80	787	22.23	1130	30.35
ABT-PPF	18.72	762	22.40	1253	39.19
AH-CNC	18.97	744	22.63	1723	56.82
HPH-CNC	18.57	594	22.16	1077	44.85

#### FE-SEM and TEM

**Fig 4:**
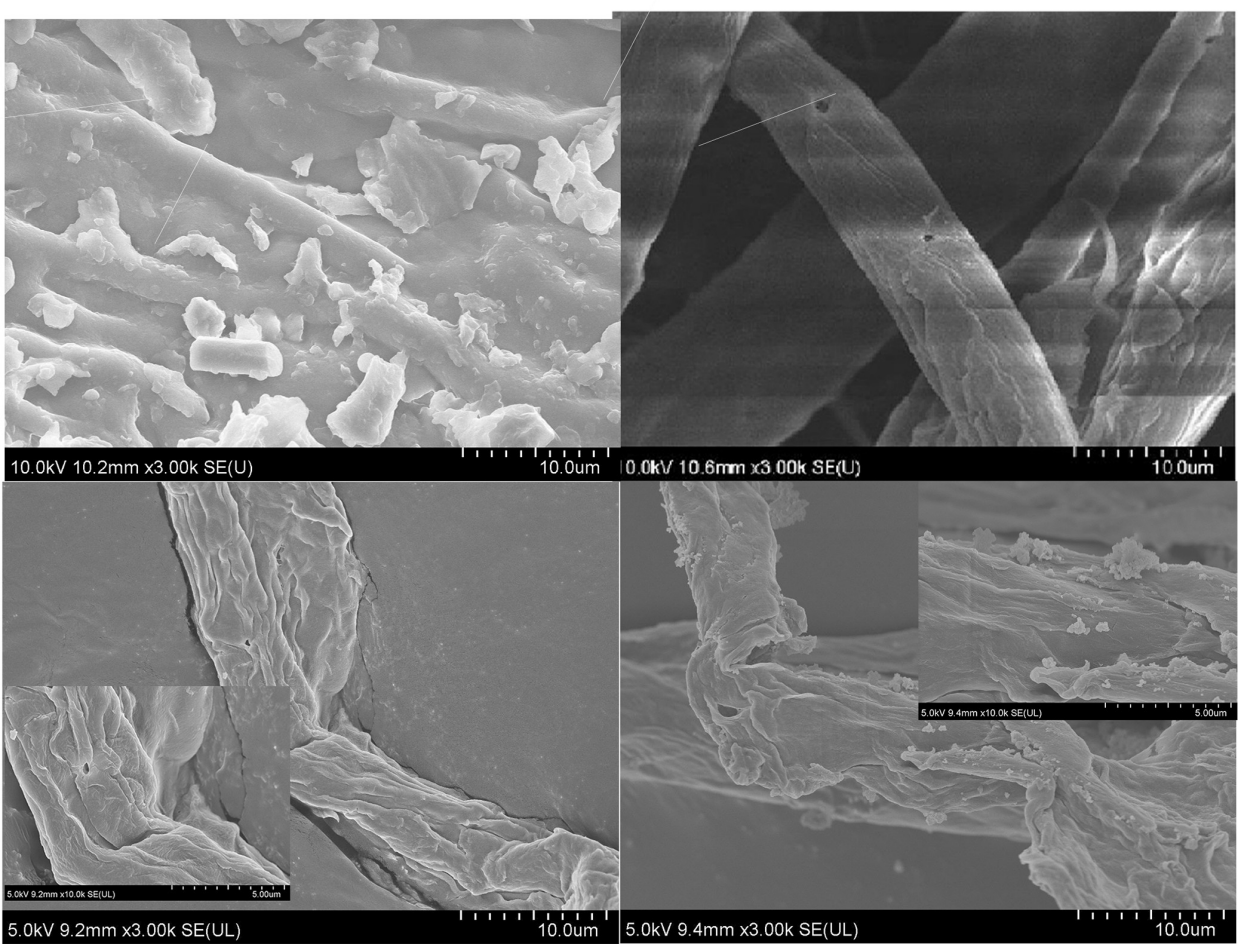
FE-SEM micrographs of (a) Raw PPF, (b) ABT-PPF, (c) AH-C and (d) HPH-CNC.

**Fig 5:**
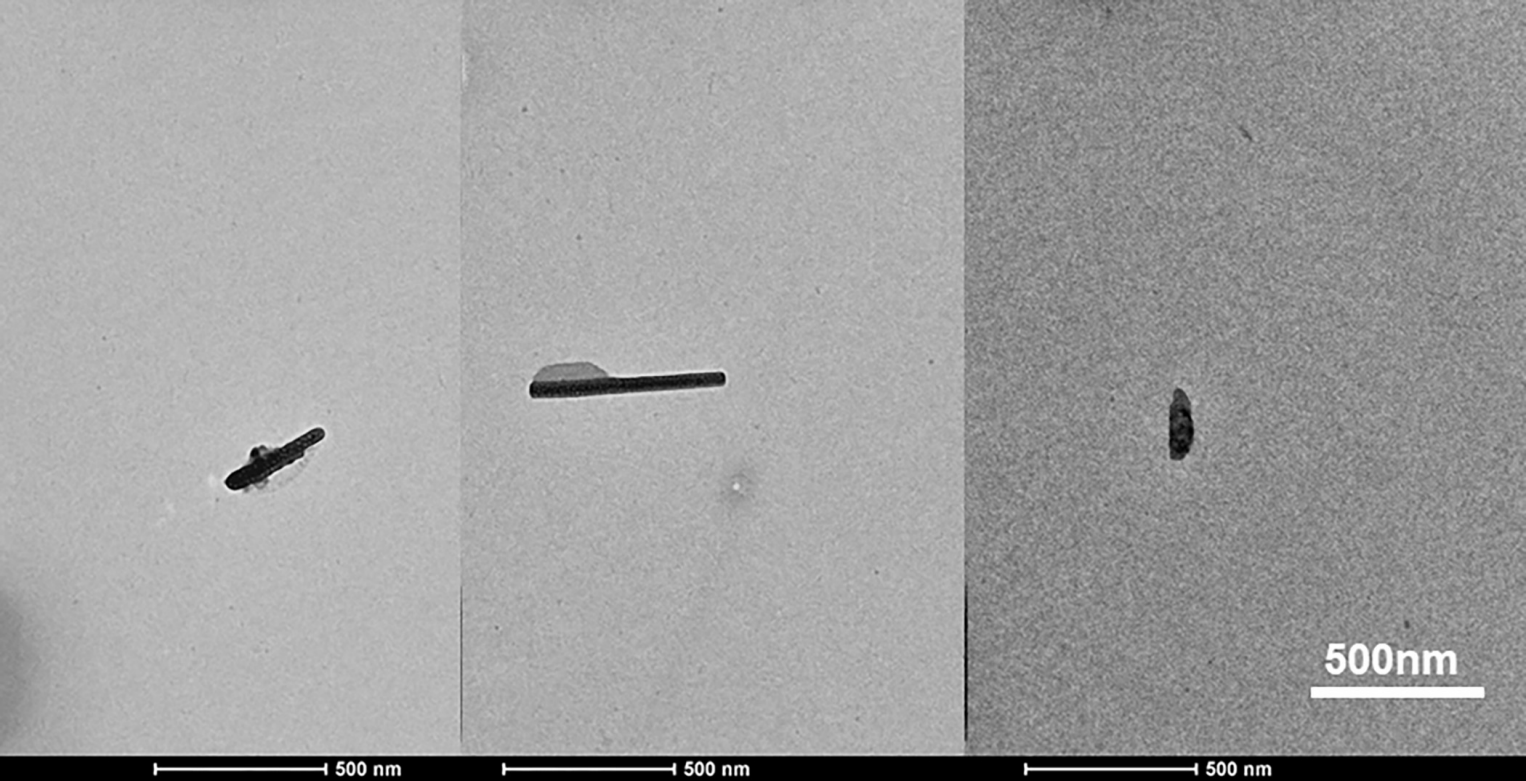
TEM micrographs of HPH-CNC (produced from 1 cycle at 50MPa).

In order to obtain an absolute measurement of particle dimension, SEM and TEM analysis were performed. Fig 4 shows the FE-SEM images of Raw PPF, ABT-PPF, AH-C, and HPH-CNC, respectively. As observed, PPF exists in the form of fibre bundles that made up of several microfibres that are held together by hemicellulose and lignin. The external surface of Raw PPF fibre bundles was rough and irregular with some protrusions on the surface due to the attachment of non-cellulosic and macromolecular substances (Fig 4A). After alkaline and bleaching treatments, separation of fibre bundles was observed. Subsequent bleaching process caused a clearer fibre surface with more fibre bundles that disintegrated into individual distinct fibres (Fig 4B). Acid hydrolysis further disintegrated the intra-fibrillar structure into flexible loosen individual cells (Fig 4C). The cellulose fibres were disintegrated and culminated in a more separate network. The CNC was more fine, smooth and tend to stick together after passing through HPH mainly because of homoegnization that induces the formation of hydrogen bonds (Fig 4D). Fig 5 shows the TEM micrograph of a dilute suspension of HPH-CNC, which reveal the rod-liked shape. TEM image further verified the CNC to be in nanosize with a length of 194.92 ± 208.40nm and a diameter of 47.85±52.91nm as well as an aspect ratio of 4.07.

The measurements of TEM and FE-SEM showed the diameter of HPH-CNC was 47.85± 52.91 nm. These values were noticeably lower than the diameter measured by Zetasizer (82.6 ± 6.3 nm for C-50P and 89.5 ± 7.5 for C-1C). Foo, et al. [51] pointed out that DLS serves as an ideal tool in measuring the hydrodynamic diameter of spherical particles. The discrepancy could be due to the limitation of DLS method when it comes to measuring the size of non-spherical particles like the rod-shaped CNC. In the case of non-spherical particles, DLS could only offer a rough estimation on the equivalent hydrodynamic size based on a spherical model, hence suggesting a slightly deviated diameter [52]. Also, the sedimentation and agglomeration of CNC particles could affect the particle size measurement, leading to overestimation. This is because the conformational change of particles has an influence on the particle diffusion rate which will correspondingly change the hydrodynamic size [52]. Therefore, the real particle size could be much smaller than the one detected by DLS technique. This was further proved by study which showed that the measurement done by DLS was mostly >90% bigger than the TEM measurement [53]. Overall, the role of HPH in CNC extraction from PPF is undeniable. HPH improves zeta potential, viscosity, and stability (separation) of the acid hydrolysed cellulose as well as making them finer.

#### FTIR

**Fig 6:**
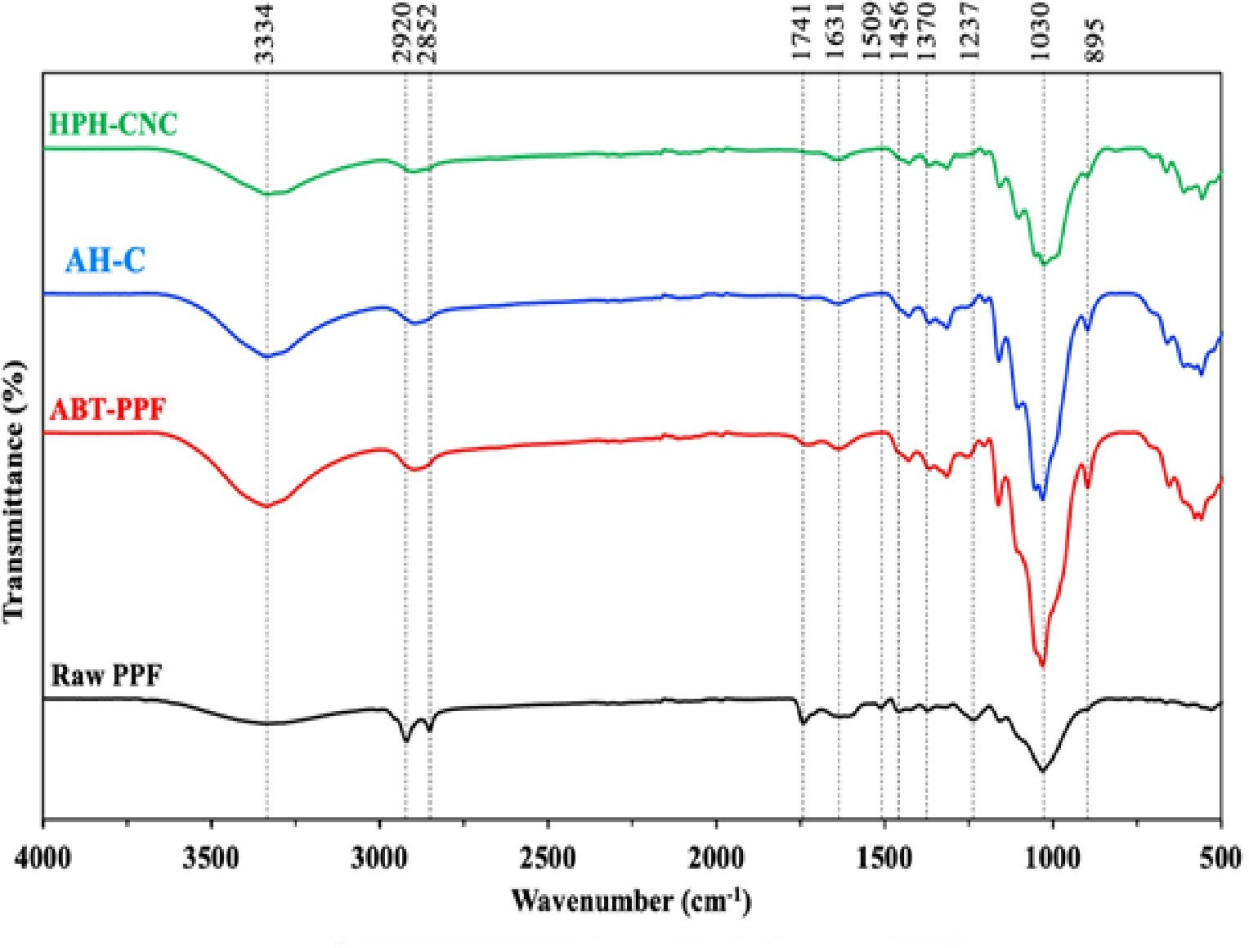
FTIR spectra of Raw PPF, ABT-PPF, AH-C and HPH-CNC.

Fig 6 depicts the FTIR spectra of Raw PPF, ABT-PPF, AH-C and PH-CNC. In Raw PPF, the prominent peak at 1741 cm^-1^ was attributed to either C = O stretching vibration of the acetyl and uronic ester groups from hemicellulose or the ester linkage of carboxylic group of ferulic and *p*-coumaric acids of lignin or hemicellulose [54]. This peak disappeared when Raw PPF was chemically treated, possibly due to the removal of hemicellulose and lignin. Another peak that appeared only in the spectrum of Raw PPF was at 1509 cm^-1^ which represented aromatic C = C vibration from the aromatic ring of lignin [11]. Diminishing of this peak indicated the effective removal of lignin. Moreover, the peak at 1237 cm^-1^ in the spectrum of Raw PPF was linked to the C–O vibration of ethers, esters and phenol groups of waxy substances, the C–O acetyl groups in hemicellulose or the C–O–C stretching of hemicellulose, lignin and cellulose [20]. This peak disappeared after treatment, confirming the elimination of hemicellulose and lignin from the Raw PPF. Acid hydrolysis process further removed the amorphous part of the cellulose fibres and liberates individual crystallites. Elimination of amorphous region was shown in the two peaks at 1030 cm^-1^ and 895 cm^-1^. The intensity of these peaks increased after chemical pre-treatments and acid hydrolysis but weakened after HPH treatment, indicating an increase in crystallinity and cellulose content of the fibres after acid hydrolysis, followed by a slight reduction in crystallinity owing to HPH treatment.

### Characterization of CNC-stabilised o/w emulsions

#### Stability of Pickering emulsion

In this section, sample C-1C was selected to stabilise emulsions because it has the smallest particle size. It also, had a highest aspect ratio and zeta potential. Despite C-7C attained a higher aspect ratio and zeta potential than C-1C, it was not selected as homogenising for 7 cycles required intensive energy. Furthermore, the gel-like behaviour of extracted CNC favour the establishment of a gel network around the oil droplets, providing a mechanical barrier towards droplet coalescence [15]. As 0.30% CNC (CNC_0.30_-PE) is sufficient to stabilise the Pickering emulsion, we further investigated the emulsification capability at a lower concentration of 0.15% CNC (CNC_0.15_-PE). Stability of the emulsion was evaluated under different pH, temperature and ionic strength over a period of 6–14 days. In the present study, the measurement of particle size of the Pickering emulsion conferred a large standard deviation. It could also be resulted from the excessive nanocelluose that was not coated on the oil droplet surface but instead distributed in the continuous phase of the emulsion.

**Fig 7:**
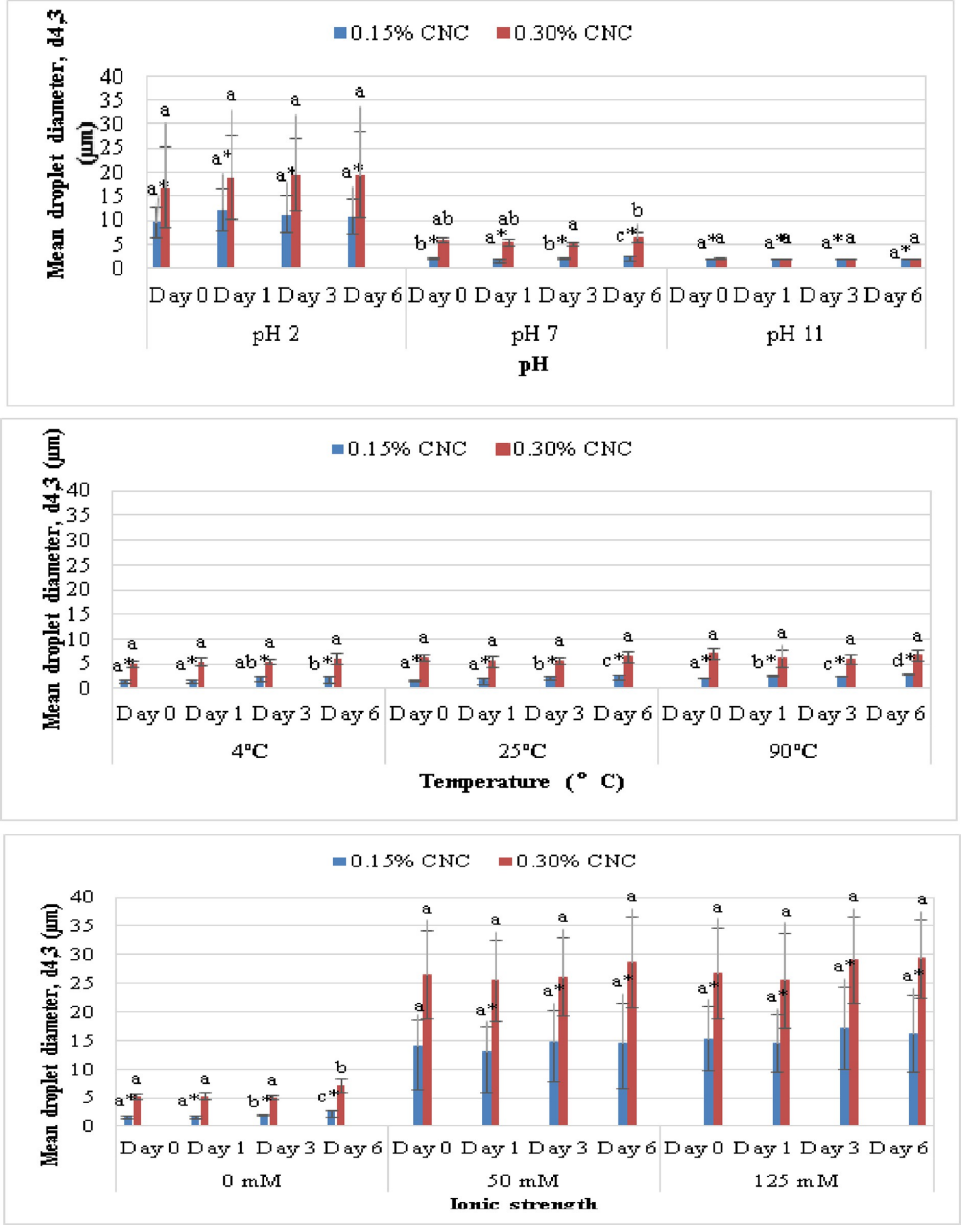
top) Mean droplet diameter, d4,3 (μm) against pH (2, 7, 11); centre) temperature (4°C, 25°C and 90°C); bottom) ionic strength (0mM, 50mM and 124mM) for CNC0.15-PE and CNC0.30-PE over 6 days of storage. ^abc^ represents values within the same conditions (concentration, pH, temperature and ionic strength) with same letters were significantly different (p < 0.05) across different days.

**Fig 8:**
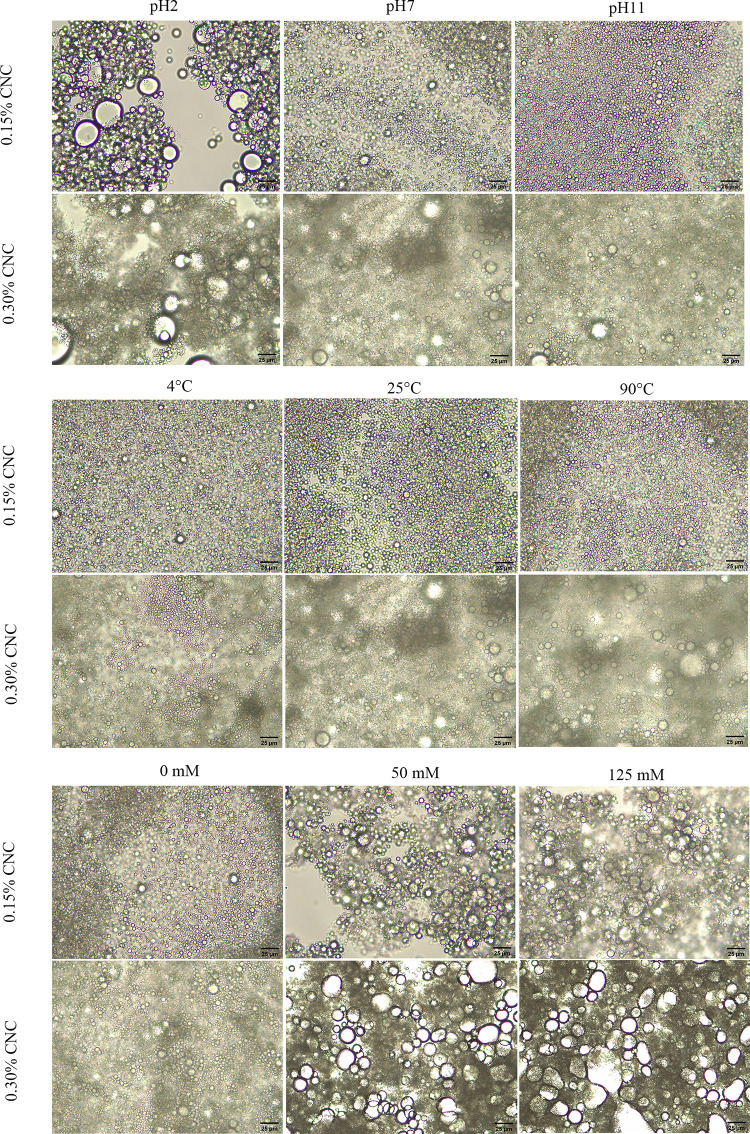
top) Optical micrographs of CNC_0.15_-PE and CNC_0.30_-PE at pH (2, 7, 11); centre) temperature (4°C, 25°C and 90°C); bottom) ionic strength (0mM, 50mM and 124mM) on Day 0 viewed under 40× magnification.

*Effect of Ph*. Stability of the emulsions as a function of pH was assessed at pH 2, pH 7 and pH 11, representing acidic, neutral and alkaline conditions, respectively. Fig 7 (top) depicts the mean droplet diameters of emulsion samples at pH 2, pH 7 and pH 11 for CNC_0.15_-PE and CNC_0.30_-PE, respectively. All emulsions exhibited a similar trend whereby the droplet diameter increased as the pH decreased. This phenomenon could be attributed to the changes in the electrostatic forces. CNC carries negative charge due to the presence of carboxyl groups (–COO^−^) on its surface [55]. Under acidic conditions (pH 2), protons from the added acid induced protonation of the negatively charged carboxyl groups (COO^−^+ H^+^ → COOH), hence reducing the net charge in the system. As a consequence, the electrostatic repulsion among CNC-coated oil droplets was weakened, leading to flocculation and coalescence and thus the enlargement of droplet size. This was in line with the observations from optical microscopy which showed aggregates of oil droplets at pH 2 as shown in Fig 8 (top). This result was in accordance with the previous research which reported an increase in the mean diameter of nanocellulose-coated lipid droplets at low pH condition [14, 55, 56].

At pH 7, emulsion showed higher stability than that of pH 2 as evidenced by the smaller droplet sizes. Under neutral conditions, many of the carboxyl groups on CNC could be ionized and the oil droplets mutually repulsed for more steric hindrance coupled with the highly attractive forces between oil and CNC particles, resulting in the stabilisation of the emulsion system [56]. Also, the superior water wetting ability or solubility of CNC at pH 7 enabled great emulsifying capacity and the formation of a stable emulsion [57].

The emulsion displayed the smallest droplet sizes at pH 11. This occurrence arose from deprotonation of some of the protonated carboxyl groups (COOH → COO^−^ + H^+^) leading to an increase in the overall surface charge. The increase in the surface charge density of CNCs improved the emulsion stability due to the enhanced electrostatic repulsion between droplets that lowered the risk of flocculation and coalescence [58]. This outcome was supported by previous research which reported decreased droplet size and enhanced emulsion stability in an alkaline environment [14, 55].

*Effect of temperature*. The effect of temperature was evaluated at 4°C, 25°C and 90°C, representing refrigeration, ambient and heating temperatures, respectively. Fig 7 (centre) portrays the mean droplet diameters of emulsion samples at 4°C, 25°C and 90°C for CNC_0.15_-PE and CNC_0.30_-PE, respectively. Majority of droplets were unaffected by thermal treatment. Similar study reported that CNC demonstrates high temperature stability with an onset thermal degradation temperature above 200°C [59]. Only a slight increase in the droplet size occurred at elevated temperature of 90°C. This corroborates with Fig 8 (centre) which showed slightly bigger droplets at 90°C. A high temperature treatment promotes kinetic mobility of CNC and reduces the rigidity of the interface, hence increasing the rate of collision between the droplets due to the higher thermal energy [60].

*Effect of ionic strength*. The effect of salt concentration (0, 50, 125 mM NaCl) on the stability of emulsion system was also investigated. Fig 7 (bottom) illustrates the mean droplet diameters of CNC_0.15_-PE and CNC_0.30_-PE at 0 mM, 50 mM and 125 mM ionic strength. CNC-stabilised emulsion is relatively stable without salt with a significantly (p < 0.05) smaller droplet size than those salt-added emulsion systems. The DLS results were well aligned with the results from optical microscopy, whereby larger droplet sizes and droplet aggregation were observed at high ionic strength (50 mM and 125 mM) for all samples, as presented in Fig 8 (bottom). The result could be explained in terms of electrostatic screening that reduces the overall net charge [61]. The addition of NaCl altered ionic strength in emulsion systems in which the Na^+^ ions screened out the negatively charged carboxyl groups on the CNC surface, thereby lowering the electrostatic repulsion between the oil droplets. Ni et al. [11] discovered that the addition of NaCl induced the electrostatic shielding of negatively charged CNC, causing a drastic increase of droplet size from 5.32 μm to 42.87 μm when the ionic strength increased from 10 mM to 200 mM.

#### Storage stability of emulsions

**Fig 9:**
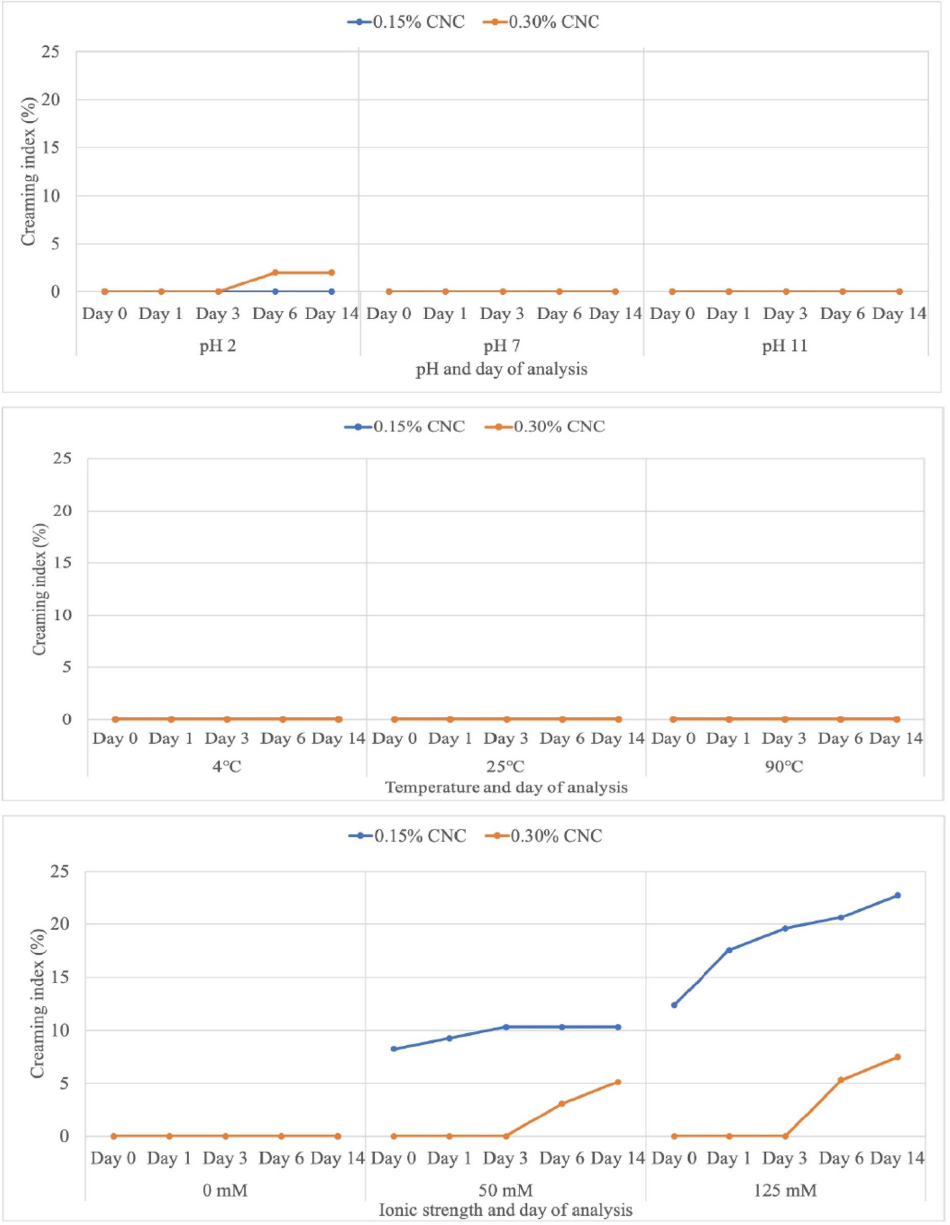
Creaming index of CNC_0.15_-PE and CNC_0.30_-PE at pH (2, 7, 11); centre) temperature (4°C, 25°C and 90°C); bottom) ionic strength (0mM, 50mM and 124mM) on Day 0, 1, 3, 6 and 14.

From a thermodynamic perspective, the emulsion system is unstable and will gradually separate into immiscible phases of oil phase, aqueous phase and emulsion phase after a period of time *via* creaming, flocculation and coalescence [62]. CI of emulsion stabilised by CNC_0.15_-PE and CNC_0.30_-PE against different pH, temperature and ionic strength was shown in Fig 9. CNC showed a negligent CI, indicating the good emulsifying performance of CNC to maintain a stable emulsion system over 14 days at a wide range of pH. However, CNC_0.30_-PE showed a thin oil layer on top of the emulsion at pH 2 starting from Day 6. The formation of a layer of oil on top of the emulsion referred to as oiling off. Meanwhile, no phase separation was observed in the emulsion samples over 14 days at 4°C, 25°C and 90°C. In other words, CNC-stabilised emulsions had extremely high stability (CI = 0) against temperature. On the other hand, the emulsions were relatively stable without the addition of salt throughout the 14 days storage. Poor creaming stability of the emulsions was observed at high salt concentration.

#### Effect of CNC concentration

A comparison of droplet diameters between two CNC concentrations revealed that CNC_0.30_-PE showed significantly greater droplet sizes than CNC_0.15_-PE at all pH, ionic strength and temperature levels. However, in comparison with CNC_0.15_-PE, CNC_0.30_-PE had higher stability against creaming at different environmental stresses despite having larger droplet sizes. The higher the concentration of CNC, the greater the stability of the emulsion system. By increasing the CNC concentration, the viscosity of the continuous phase increases which play an important role in improving the creaming stability of emulsion as stated by Stoke’s law [63]. As such, the movements of oil droplets are restricted, leading to an interfacial tension reduction and offering stronger multiple layers in preventing droplet coalescence [64]. In addition, the great stability of emulsion stabilised by higher CNC concentration could be attributed to the depletion-flocculated network in which a three-dimensional network of droplets and CNC particles is formed in the presence of non-adsorbing excess CNC particles [63]. This viscoelastic network immobilises the droplets, thereby preventing the coalescence of oil phase and mechanically stabilising the system.

## Conclusion

In this study, CNC was successfully isolated from PPF by acid hydrolysis coupled with HPH treatment. HPH processing further reduces the size of acid hydrolysed CNC and improved its stability. CNC particle size decreased on account of an increase in the homogenisation pressure but shifted slightly to a higher value with the increase in homogenisation cycle. Interestingly, HPH rendered CNC particles with a high zeta potential value of around -60 mV. Emulsion stabilised by CNC demonstrated good thermal stability. However, the stability of emulsion was slightly lower at low pH and at high salt concentration. All the emulsion incorporated with CNC_0.15_-PE and CNC_0.30_-PE alone are sufficient to stabilise emulsion. Nevertheless, CNC_0.30_-PE showed to have greater stability against creaming at different environmental stresses as compared to CNC_0.15_-PE. In conclusion, PPF is deemed as an appropriate and sustainable cellulosic source for isolation of CNC. The extraction method using high energy emulsification route is able to improve acid hydrolysed CNC with extremely high zeta potential which is an essential characteristic for stabilising emulsion. Taken together, CNC isolated has great potential to be used as natural stabiliser for oil-in-water food emulsion to replace the synthetic stabiliser.

## Supporting information

S1 File (ZIP)

S1 Graphical abstract (JPG)
