# Supplementary figures and images for: Pickering emulsion stabilized by palm-pressed fiber cellulose nanocrystal extracted by acid hydrolysis-assisted high pressure homogenization
Source: PLoS One. 2022 Aug 31;17(8):e0271512. doi: 10.1371/journal.pone.0271512 (PMC9432738; doi:10.1371/journal.pone.0271512)

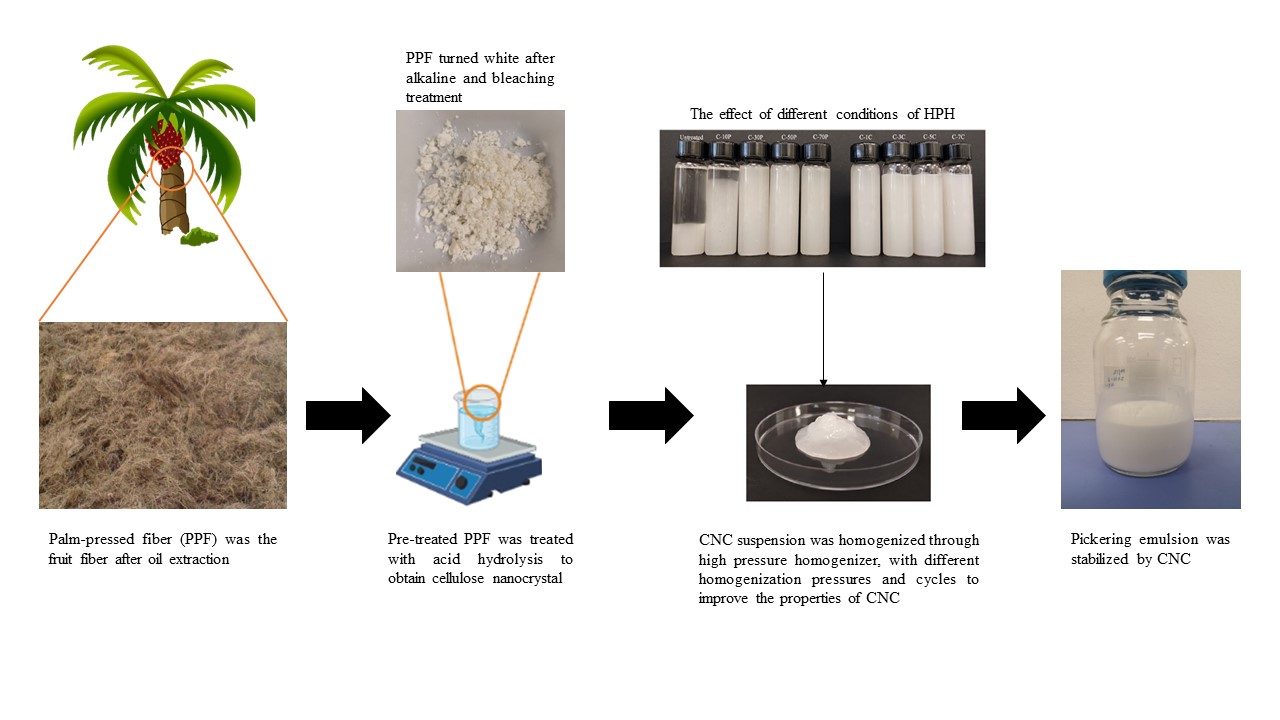

Supplement: S1 Graphical abstract — (JPG) [file pone.0271512.s002.jpg]
